# OP36 Real-World Data From Early Access Programs In France: A Two-Year Review

**DOI:** 10.1017/S0266462324000989

**Published:** 2025-01-07

**Authors:** Camille Thomassin, Judith Fernandez, Margaret Galbraith, Céleste Babin, Marion Bardin, Thierno Diatta, Alice Desbiolles, Alexandre Beaufils, Floriane Pelon, Pierre Cochat

## Abstract

**Introduction:**

The 2021 reform of early access (EA) for medicinal products in France includes a mandatory data collection for each patient. The objective of this work was to perform a two-year review of the protocols for therapeutic use and data collection (PUT-RD) validated by the health technology assessment body (Haute Autorité de Santé [HAS]) in collaboration with the national regulatory body where applicable.

**Methods:**

The minimum dataset defined in the PUT-RD varies depending on the regulatory status of the product and may include patients’ characteristics, conditions of use, efficacy, and safety data. A descriptive review of the PUT-RDs approved between 1 July 2021 and 30 June 2023 was carried out by HAS. The following information was retrieved in each published PUT-RD: type of EA (pre- or post-marketing authorization); collection of efficacy data (yes or no) (if yes, inclusion of a patient-reported outcome measure [PROM] or no); type of data source (registry or electronic platform provided by a contract research organization [CRO]).

**Results:**

During the review period, a total of 98 PUT-RDs were validated, corresponding to 98 EA favorable decisions. EAs were authorized prior to marketing authorization in 40 percent of the cases. A collection of follow-up efficacy data was planned in 52 PUT-RDs, including integration of patients’ perspectives through a PROM in 32 PUT-RDs. Data collection through a registry was planned in six PUT-RDs (using the DESCAR-T registry), otherwise data collection was mainly planned through private electronic platforms operated by CROs (80% of the PUT-RDs).

**Conclusions:**

To optimize the generation of relevant clinical data to be used in the regular HTA process, EAs should be requested early, prior to marketing authorization. Additionally, the use of existing data sources, in particular disease registries, is to be developed to avoid duplicate data collection and alleviate the burden for healthcare professionals.

